# A Generic Mechanism of Emergence of Amyloid Protofilaments from Disordered Oligomeric Aggregates

**DOI:** 10.1371/journal.pcbi.1000222

**Published:** 2008-11-14

**Authors:** Stefan Auer, Filip Meersman, Christopher M. Dobson, Michele Vendruscolo

**Affiliations:** 1 Centre for Self Organising Molecular Systems, University of Leeds, Leeds, United Kingdom; 2 Department of Chemistry, Katholieke Universiteit Leuven, Leuven, Belgium; 3 Department of Chemistry, University of Cambridge, Cambridge, United Kingdom; National Cancer Institute United States of America and Tel Aviv University Israel

## Abstract

The presence of oligomeric aggregates, which is often observed during the process of amyloid formation, has recently attracted much attention because it has been associated with a range of neurodegenerative conditions including Alzheimer's and Parkinson's diseases. We provide a description of a sequence-indepedent mechanism by which polypeptide chains aggregate by forming metastable oligomeric intermediate states prior to converting into fibrillar structures. Our results illustrate that the formation of ordered arrays of hydrogen bonds drives the formation of *β*-sheets within the disordered oligomeric aggregates that form early under the effect of hydrophobic forces. Individual *β*-sheets initially form with random orientations and subsequently tend to align into protofilaments as their lengths increase. Our results suggest that amyloid aggregation represents an example of the Ostwald step rule of first-order phase transitions by showing that ordered cross-*β* structures emerge preferentially from disordered compact dynamical intermediate assemblies.

## Author Summary

Considerable efforts are currently devoted to the study of the phenomenon of protein aggregation because of its association with a wide variety of human diseases and of its potential applications in biotechnology. Despite intense scrutiny, however, it has been extremely challenging so far to characterise in detail the process by which peptides and proteins aggregate. We have used here molecular simulations to show that the growth of ordered structures from initially disordered assemblies is a consequence of the interplay between two fundamental interactions common to all proteins—hydrophobicity and hydrogen bonding. These results provide further insight into the consequences of the “generic hypothesis” of protein aggregation, according to which the ability to assemble into ordered structures is not an unusual feature exhibited by a small group of peptides and proteins with special sequence or structural properties, but it is an inherent characteristic of polypeptide chains.

## Introduction

A variety of peptides and proteins unrelated in sequence and structure have been shown to convert into large ordered aggregates known as amyloid fibrils [1],[2]. These structures share a common cross-*β* structure formed by intertwined layers of *β*-sheets extending in a direction parallel to the fibril axis [1],[3]. The ubiquity of this type of assemblies has led to the suggestion that they may represent a general structural state of polypeptide chains, which is accessible independently from their specific amino acid sequences [4]. According to this view, if placed under appropriate conditions, peptides and proteins can revert to the amyloid state, which has been associated with a range of pathological conditions including Alzheimer's and Parkinson's diseases [1],[5],[6].

Small oligomeric aggregates are often found as precursors of amyloid fibrils [7]–[9], and their formation in some cases may originate from a competition between amorphous and fibrillar aggregation. The role of these molecular species in the process of amyloid fibril formation is at present unclear, although much interest has been recently devoted to this problem since their presence has been linked to neurodegenerative processes [8],[10]. It has been suggested that, under conditions that favor amyloid fibril formation, proteins or peptides within these disordered aggregates can convert into conformations capable of forming nuclei that give rise to amyloid fibril assemblies [9]. It has been, however, extremely challenging to characterize experimentally the structures of these aggregates and the mechanism of their formation owing to their heterogeneous and dynamical nature.

In this work we use computer simulations to describe the process of condensation of polypeptide chains into oligomeric assemblies that further reorganise into fibrillar structures. The level of detail in which protein aggregation can be investigated depends on the choice of the model. Full-atomistic simulations have provided considerable insight into the dynamics of inter-molecular interactions in systems containing a small number of peptides and short timescales [11]–[17]. Complementary to these approaches, coarse-grained models have proven capable of simulating larger systems and longer timescales, and of following the structure of the oligomeric intermediates and the mechanism of their conversion into ordered cross-*β* assemblies [18]–[22]. Despite much recent work in this area, many questions about the amyloid aggregation remain open, and here we investigate the general properties of the mechanism of emergence and alignment of *β*-sheets in the early stages of the oligomerization process. Given the close link between this phase of amyloid formation and the neurotoxicity of the structural species involved [1],[8],[10],[23], we investigated here the competition between ordered and disordered aggregation of polypeptide chains.

By following the hypothesis that amyloid formation represents a generic property of a polypeptide chain [4], we adopt a recently proposed representation of polypeptide chains, known as the tube model [24]–[27]. This model enables a description of the free energy landscapes for folding [24],[25],[27] and for aggregation [26],[28] to be obtained within a unified framework by capturing the intrinsic symmetry of polypeptide chains, which is shown to be able to create by itself conformations with protein-like topologies both in the monomeric and in the multimeric forms [24]–[28]. Since the version of the tube model that we used in this work only includes interactions common to all polypeptide chains independently from their amino acid sequence, it is ideally suited for exploring the consequences of the generic hypothesis of amyloid formation. The characteristic features of the model [24],[26] are that the protein backbone is assigned a finite thickness to account for excluded volume effects. Residues interact with each other by pairwise additive hydrophobic forces (with energy *e_W_*), geometrical constraints apply to the formation of intra- and intermolecular hydrogen bonds (with energy *e_HB_*), and the polypeptide chain experiences a local bending stiffness (with energy *e_S_*).

## Results

**Figure 1:**
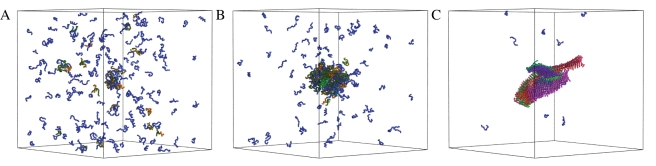
Illustration of the self-assembly process of peptides into amyloid-like assemblies. All simulations were carried out at a concentration *c* = 12.5 mM and reduced temperature T* = 0.66. The progress variable *t* corresponds to the number of Monte Carlo moves performed in the simulation, and one unit of *t* is a series of 10^5^ Monte Carlo moves. Initially, at *t* = 1000 (A), all peptides are in a solvated state. As the simulation progresses, at *t* = 5000 (B), a hydrophobic collapse causes the formation of a disordered oligomer, which subsequently undergoes a structural reorganization into an amyloid-like assembly, at *t* = 30 000 (C), driven by the formation of ordered arrays of hydrogen bonds. Peptides that do not form intermolecular hydrogen bonds are shown in blue, while peptides that form intermolecular hydrogen bonds are assigned a random color, which is the same for peptides that belong to same *β*-sheet.

In this work we consider a system containing 216 12-residue homopolymers that exibit an *α*-helical native state below the folding temperature (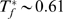) and an undfolded structure at higher temperatures (see Materials and Methods for the definition of the temperature scale used here). Peptides that form native *α*-helical conformations [29], as well as homopolymeric sequences [30], have been shown to be able to form amyloid assemblies. In order to investigate the self-assembly of the peptides into fibrils we chose thermodynamic conditions such that fibril formation occurs on a timescale accessible to our simulations. We found that a peptide concentration *c* = 12.5 *mM* is above the critical concentration for aggregation, for temperatures below *T* ^*^ = 0.69. All our simulations were performed at *T* ^*^ = 0.66, and several independent starting configurations were generated at *T* ^*^ = 0.75. As in our simulations we set 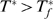, the peptides were unfolded most of the time. A typical trajectory observed in our Monte Carlo simulations (see Materials and Methods) is illustrated in Figure 1.

**Figure 2:**
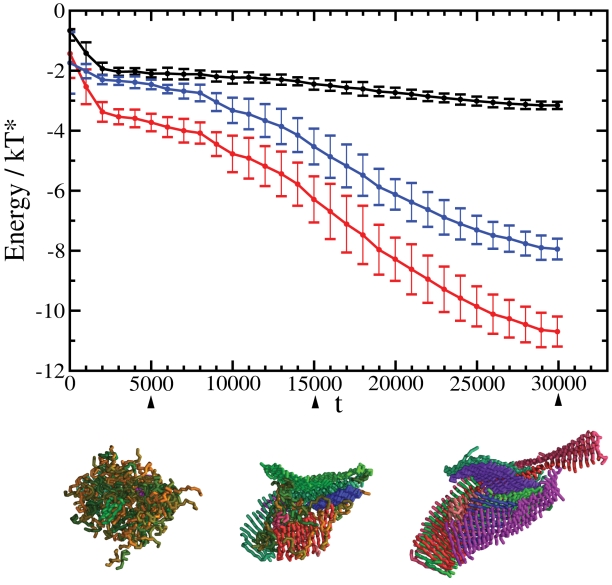
Time series of the energy per peptide as a function of the progress variable (*t*). Together with the total energy (red line), we show the contributions from the hydrogen bonding energy (blue line), and the hydrophobic energy (black line). The gradual emergence of the cross-*β* ordering from the initially disordered oligomeric assemblies is characterised by a significant increase in the weight of the hydrogen bonding energy. Errorbars represent standard deviations over 11 independent trajectories. Representative structures formed during the process of conversion of the disordered oligomer into an amyloid-like structure are also shown at *t* = 5000, *t* = 15 000, and *t* = 30 000. The color code is as in Figure 1.

We systematically observed a rapid collapse of the peptides into disordered aggregates that subsequently underwent a structural reorganization and transform into cross-*β* protofilaments (Figure 1). These results are consistent with a previously described two-step condensation-ordering mechanism [16],[18],[28], which has also been observed experimentally [9]. A plot of the total energy per peptide as a function of the progress variable *t* (Figure 2) shows that the final structure has a much lower energy than the initial and intermediate states. The major contribution to this energy comes from hydrogen bonding (Figure 2), a result consistent with the recent report that the hydrogen bonding energy provides the dominant factor stabilising the cross-*β* architecture is represented by hydrogen bonding, while in more disordered states other contributions are also important [31]. The initial state (*t*<1000), before the hydrophobic collapse, in which all peptides are solvated, has the highest energy and it is unstable. After the hydrophobic collapse has taken place (1000<*t*<5000), the peptides form a disordered oligomer, which is characterised by similar contributions from hydrophobic interactions and hydrogen bonding (Figure 2); this oligomeric state is lower in energy but metastable with respect to the amyloid state. Finally, with the growth of the cross-*β* architecture the hydrogen bonding interactions become progressively dominant (Figure 2). The survival time of the disordered oligomeric state is rather short (about 10–15% of the total simulation time) since in order to be able to investigate the self-assembly of the peptides we chose thermodynamic conditions such that the nucleation barriers associated with oligomer formation and the subsequent ordering are readily overcome by thermal fluctuations. The height of the nucleation barriers, and the associated lag times depend strongly on the thermodynamic conditions of the system [28].

In order to provide a detailed description of the emergence of cross-*β* protofilaments within the oligomers, including their interactions and relative orientations with respect to each other, we defined the oligomeric state using a distance criterion that requires the centres of mass of two peptides to have a distance of less than 5*Å*. Two peptide chains are taken to form a *β*-sheet if they share more than four inter-chain hydrogen bonds with each other. To define an angle between different *β*-sheets we calculated the relative orientation between neighboring peptides that constitute the different *β*-sheet. Therefore we calculate the dot product of the end to end vectors of the peptide molecules, requiring that the centres of mass of two peptides are separated by less than 10*Å*, which is the typical inter-sheet contact distance in most native and amyloid systems [1]. If the average angle between two *β*-strands is less than 20 degree, we assume that the respective *β*-sheets belong to the same protofilament.

**Figure 3:**
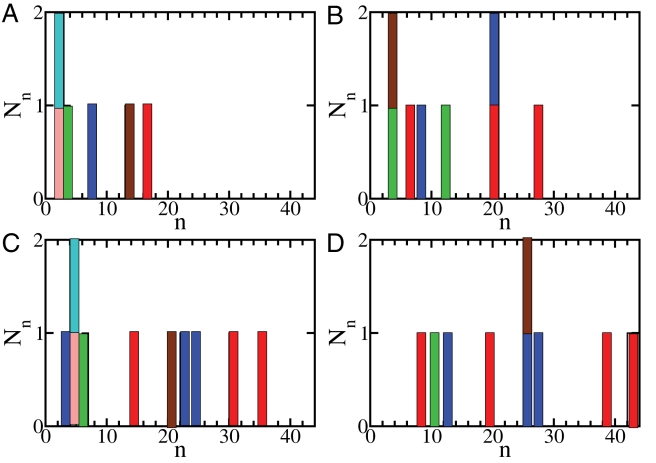
Histogram of the number *N_n_* of *β*-sheets consisting of *n* peptides at four successive stages of the growth and reordering process of the oligomeric assembly shown in Figure 1: (A) *t* = 10 000, (B) *t* = 15 000, (C) *t* = 20 000, (d) *t* = 30 000). This plot shows how *β*-sheet assemblies are progressively formed by the growth and alignment of individual *β*-sheets. At *t* = 10 000 (A) there are six *β*-sheets of sizes ranging from 3 to 16, whereas at *t* = 30 000 (D), there are nine *β*-sheets of sizes ranging from 8 to 42. If *β*-sheets are aligned so that the angle between them is smaller than 20 degrees, they are considered to form a protofilament-like structure, and the corresponding bars in the histogram are shown with the same color, as for instance in the case of the red assembly (Figure 1c, right), formed by four *β*-sheets of size 8, 19, 38, and 42.

In the example illustrated in Figure 1, the initial stages of the process are characterized by the formation within the disordered oligomer of six small *β*-sheets that are randomly oriented with respect to each other (Figure 3a). Subsequently, the *β*-sheets tend to align as their lengths increase, and protofilaments consisting of one, three and four *β*-sheets are formed (Figure 3b–d). The two major protofilaments observed in this simulation seem to twist around each other (Figure 1, right), resembling the typical behavior observed experimentally [1]. The twisting appears to follow from the growth and alignment of *β*-sheets, which is a consequence of the tendency to optimize the number of hydrophobic contacts, thereby reducing the interfacial energy [32], and not from the chirality of the peptides, as the latter is not included in the tube model used in this work. As the peptides within the oligomer can move only locally our Monte Carlo dynamics should at least qualitatively resemble their actual dynamics.

**Figure 4:**
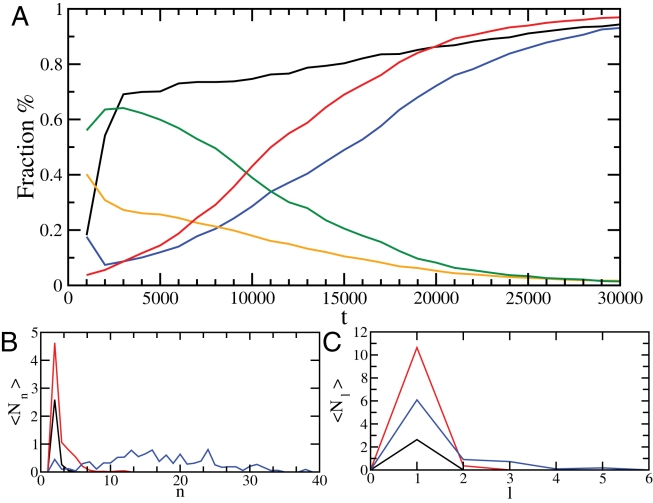
Analysis of the evolution of the structure of the oligomers over 11 independent simulations. (A) Development of the fraction of polypeptide chains in a oligomer (black), fraction of polypeptide chains in a oligomer that form a *β*-sheet conformation (blue), fraction of hydrogen bonds in a oligomer in a *α*-helical conformation (orange), and in a *β*-sheet conformation (red), or otherwise (green). (B) Development of the distribution function of the average number of *β*-sheets 〈*N_n_*〉 of size *n* at *t* = 1000 (black), *t* = 5000 (red), *t* = 30 000 (blue). (C) Distribution function 〈*N_l_*〉 of the number of protofilaments composed of *l* layers at *t* = 1000 (black), *t* = 15 000 (red), *t* = 30 000 (blue).

We generated and analyzed a total of 11 independent trajectories, which consistently appeared as the type shown in Figure 1, and showed the same quantitative overall behavior. Assemblies are initially formed through the disordered rapid assembly of partially folded peptides, which then reorganize into ordered *β* sheets. A quantitative analysis (Figure 4) of the reordering process shows that initially about 60% of the hydrogen bonds within the oligomers are formed in disordered intermolecular associations, whereas the remainder are involved in intramolecular interactions within the native *α*-helix conformation (Figure 4a). At later stages, a structural reorganization of the oligomers results in essentially all hydrogen bonds being involved in the cross-*β* structure. Thus, in agreement with experimental evidence [33]–[35], we found that the formation of disordered oligomers is primarily driven by hydrophobic effects, whereas a reorganisation driven by hydrogen bond formation is subsequently playing a major role in the formation of cross-*β* structure [16],[28]. The formation of ordered assemblies starts with the pairing of two peptides, from which larger *β*-sheets develop (Figure 4b). As the simulation progresses, the height of the peak in the size distribution function associated with single *β*-sheets decreases and multi-layer *β* sheets form, thus revealing the process of protofilament formation (Figure 4c). This observation complements and extends the analysis shown in Figure 3, which shows that the *β* sheets align as they grow in size.

## Discussion

Although the presence of disordered aggregates might not always be a prerequisite for amyloid fibril formation, these aggregates do seem to appear as intermediate states in many cases, and indeed it has been suggested that in some instances they may serve as initiation sites for amyloid fibril growth [36],[37]. The simulations that we present provide molecular details of a sequence-independent mechanism of formation of amyloid-like structures from the initial disordered aggregates. This mechanism depends on the interplay between hydrophobic forces that favor an amorphous collapse and hydrogen bonding that favor the formation of the ordered cross-*β* structure characteristic of amyloid fibrils. The *β*-sheets that form within disordered oligomers tend to align into protofilaments, which then can twist around each other as their lengths increase. In many protein systems this mechanism will be modulated by the presence of additional interactions, such as steric repulsions or side chain hydrogen bonding, which are highly sequence specific, but the results that we present show that such a mechanism can emerge as a generic feature common to all polypeptide chains. This phenomenon thus appears to be an example of the Ostwald step rule in first order phase transitions [38] in which the metastable intermediate phase from which nucleation takes place is represented by the disordered compact and highly dynamical oligomeric assemblies that form prior to the establishment of the ordered cross-*β* amyloid structure. The general nature of this type of mechanism thus provides a rationalisation of the observation that oligomeric assemblies appear to share common structural features, including those that enable them to bind to the same antibodies independently from the sequences of their constituent peptides and proteins [39].

In summary, in this work we have investigated the consequences of the generic hypothesis of amyloid formation [4] by adopting a model of protein structure specifically designed to capture the characteristic of polypeptide chains that are common to all peptides and proteins [24]. Our results have provided further support to the view that the presence of partially ordered oligomeric assemblies of the type associated with neurotoxicity constitutes a generic aspect of the phenomenon of polypeptide aggregation.

## Materials and Methods

### Description of the Model

The tube model only considers interactions that are common to all polypeptide chains, and does not include biases towards specific configurations. In the model [24] each residue is represented by a *C_α_* atom. The atoms are connected into a chain (the protein backbone) with a fixed distance of 3.8Å between neighboring atoms. The lines joining the *C_α_* atoms constitute the axes of hard spherocylinders (cylinders capped by hemispheres) of diameter 4Å. Spherocylinders that do not share a *C_α_* atom are not allowed to interpenetrate. Bond angles are restricted between 82° to 148°, and bending stiffness is introduced by an energetic penalty, *e*_S_,>0 for angles less than 107.15°; these are the same criteria used in the original formulation of the tube model [24]. Hydrophobicity enters through a pairwise-additive interaction energy of *e*_H*P*_ (positive or negative) between any pair of residues *i* and *j*>*i*+2 that approach closer than 7.5Å.

The cylindrical symmetry of the tube is broken by the presence of hydrogen bonds. A hydrogen bond has an energy *e*_H*B*_<0 and is considered to exist between a pair of residues when the two normal vectors defined by each *C_α_* atom and its two neighbors are mutually aligned to within 37° and at the same time each of these vectors lies within 20° of the vector joining the *C_α_* atoms. These geometrical requirements were deduced from a study of native protein structures [24]. There is also a distance criterion, which is different for local hydrogen bonds (between residues *i* and *j* = *i*+3), and non-local (*j*>*i*+4) hydrogen bonds. No more than two hydrogen bonds per residue are permitted, and the first and last *C_α_* atom cannot form inter-chain hydrogen bonds. Hydrogen bonds may form cooperatively between residues (*i*, *j*) and (*i*+1, *j*+1), thereby gaining an additional energy of 0.3*e*_HB_. For details of the distance and angle criteria, the reader is referred to Table 1 of the original article on the tube model [24].

To set the energy scale of the model, the energy of a hydrogen bond is fixed in all simulations at *e*_HB_ = −3*kT_o_*, where *kT_o_* is a reference thermal energy and *k* is Boltzmann's constant. This value corresponds approximately the energy associated with a hydrogen bond (1.5 kCal/mol at room temperature [40]). Values of the hydrophobicity and stiffness parameters *e*_HP_ and *e*_S_ are given in units of *kT_o_* and the reduced temperature is *T* ^*^ = *T*/*T_o_*. In all our simulations we set *e_S_* = 0.9 and *e_HP_* = −0.15. The ratio of a hydrogen bonding energy to hydrophobic energy is a parameter that we set to *e*_HB_/*e*_HP_ = 20, which is a value commonly used in simulations of the aggregation process [18],[20]. As the number of hydrophobic contacts in compact disordered phases usually about one order of magnitude larger than the number of hydrogen bonds, our choice ensures that these interactions can contribute in a similar manner to the energy of the system.

### Simulation Techniques

We performed Monte Carlo simulations in the canonical ensemble using crankshaft, pivot, reptation, displacement and rotation moves [28]. To reduce finite size effects we used a cubic box and applied periodic boundary conditions. In order to analyze the structure of the oligomers we used a distance criterion to define a disordered oligomer, which requires two peptides to have a distance of less than 5 Å. Two peptide chains are considered to form a *β*-sheet if they have more than four inter-chain hydrogen bonds with each other. To define an angle between different *β*-sheets we calculated the relative orientation between neighboring peptides that constitute the different *β*-sheet. Therefore we require that the centers of mass of two peptides are separated by less than 10*Å*, which is the typical inter-sheet distance in both native and most amyloid systems [1]. To extract the angle we calculate the dot product of the end to end vectors of the peptide molecules. If the average angle between two *β*-strands is less than 20 degrees, we assume that the respective *β*-sheets belong to the same protofilament.
